# Clinical, genetic, and sociodemographic predictors of symptom severity after internet-delivered cognitive behavioural therapy for depression and anxiety

**DOI:** 10.1186/s12888-025-07012-x

**Published:** 2025-05-30

**Authors:** Olly Kravchenko, Julia Bäckman, David Mataix-Cols, James J. Crowley, Matthew Halvorsen, Patrick F. Sullivan, John Wallert, Christian Rück

**Affiliations:** 1 Centre for Psychiatry Research, Department of Clinical Neuroscience, Karolinska Institutet & Stockholm Health Care Services, Region Stockholm, Sweden; 2 Karolinska University Hospital Huddinge, Region Stockholm, M48, SE-14186 Sweden; 3 Department of Clinical Sciences, Lund University, Lund, Sweden; 4 Department of Genetics, University of North Carolina at Chapel Hill, Chapel Hill, USA; 5 Department of Medical Epidemiology and Biostatistics, Karolinska Institutet, Stockholm, Sweden

**Keywords:** Depression, Anxiety, Cognitive behavioural therapy, Treatment outcome

## Abstract

**Background:**

Internet-delivered cognitive behavioural therapy (ICBT) is an effective and accessible treatment for mild to moderate depression and anxiety disorders. However, up to 50% of patients do not achieve sufficient symptom relief. Identifying patient characteristics predictive of higher post-treatment symptom severity is crucial for devising personalized interventions to avoid treatment failures and reduce healthcare costs.

**Methods:**

Using the Swedish multimodal database MULTI-PSYCH, we evaluated novel and established predictors associated with treatment outcome and assessed the added benefit of polygenic risk scores (PRS) and nationwide register data in a sample of 2668 patients treated with ICBT for major depressive disorder, panic disorder, and social anxiety disorder. Two linear regression models were compared: a baseline model employing six well-established predictors and a full model incorporating six clinic-based, 32 register-based predictors, and PRS for seven psychiatric disorders and traits. Predictor importance was assessed through bivariate associations, and models were compared by the variance explained in post-treatment symptom scores.

**Results:**

Our analysis identified several novel predictors of higher post-treatment severity, including comorbid ASD and ADHD, receipt of financial benefits, and prior use of psychotropic medications. The baseline model explained 27%, while the full model accounted for 34% of the variance.

**Conclusions:**

The findings suggest that a model incorporating a broad array of multimodal data offered a modest improvement in explanatory power compared to one using a limited set of easily accessible measures. Employing machine learning algorithms capable of capturing complex non-linear associations and interactions is a viable next step to improve prediction of post-ICBT symptom severity.

**Clinical trial number:**

Not applicable.

**Supplementary Information:**

The online version contains supplementary material available at 10.1186/s12888-025-07012-x.

## Introduction

Major depressive disorder (MDD), panic disorder (PD), and social anxiety disorder (SAD) are highly prevalent mental health disorders [1]. MDD ranks as the first and anxiety disorders as the sixth leading cause of disability globally, measured by years lived with disability [2]. Cognitive behavioural therapy (CBT) is an effective first-line psychotherapeutic treatment for mild to moderate depression and anxiety disorders [3], [4]. To improve accessibility, CBT is increasingly delivered in a therapist-guided internet-based format (ICBT) which has shown comparable efficacy [5], [6], [7], [8], [9], [10], superior accessibility for some patients [6], and a high probability of cost-effectiveness [11], [12]. However, CBT/ICBT is not universally efficacious, with up to 50% of patients not responding sufficiently to the treatment [13], [14], [15]. Therefore, it is crucial to identify reliable predictors of unfavourable treatment outcome and develop accurate predictive models that would assist clinicians in selecting appropriate, tailored care for these patients.

Numerous clinical characteristics have been investigated as potential predictors of differential response to CBT. Baseline symptom severity is consistently shown to be the most robust predictor, but the direction of this association is unclear. Some studies suggest that a higher baseline symptom level is predictive of a poorer prognosis following treatment [13], [16], [17], [18], [19], [20]. By contrast, others report greater/faster improvement in this patient group [21], [22], [23], [24], [25], which may be explained by regression toward the mean (i.e., extreme values sampled from a random variable are likely to be closer to its mean with repeated sampling) [26]. Comorbidity with other mood and anxiety disorders [13], [19], [27], [28], [29] as well as personality disorders [27], [30], [31], [32], early onset of symptoms [17], [27], [33], [34], and family history of psychopathology [20], [21] have also been identified as potential predictors of poorer response to CBT. Furthermore, several treatment-related variables have been linked to the outcome, such as treatment expectancy [29], adherence [25], [28], [31], homework completion [35], and working alliance [36]. Lastly, among sociodemographic patient characteristics, being employed [20], [28], [31], [32], [33], married [23], and having higher education [22] are predictive of a lower post-treatment symptom severity.

Genetic differences are implicated in a wide range of individual variation in human complex traits, including differential susceptibility and response to environmental stimuli. A new area known as therapygenetics views therapeutic interventions as such a stimulus, providing the basis for the hypothesis that genetic variation could, at least partially, explain treatment effect heterogeneity [37]. Several studies suggest that polygenic risk scores (PRS) can be used to predict response to pharmacological treatments of psychiatric disorders [38], [39], [40]. PRS are constructed as a weighted sum of risk alleles of single nucleotide polymorphisms (SNPs) using summary statistics from genome-wide association studies (GWAS). Preliminary evidence suggests a link between a higher polygenic loading for autism spectrum disorder (PRS ASD) and a poorer treatment response in patients undergoing ICBT for MDD [41]. In another study using the same data as [41] with machine learning modelling, higher PRS MDD and PRS for intelligence (PRS IQ) weakly predicted remission in ICBT for MDD [42]. However, the largest therapygenetic GWA meta-analysis to date (*n* = 2724) did not identify any common genetic variants associated with the CBT treatment outcome for depression and anxiety [43], consistent with a previous GWAS that had a similar sample size and was also likely underpowered [44].

Despite the abundance of literature on predictors of treatment outcome, there are some limitations that this study aimed to address. Most existing studies are based on a relatively small number of clinical predictors that are derived from secondary data analysis of randomized controlled trials (RCT), which are costly to conduct and rely on small samples of participants who are not representative of the broader patient population, thus providing inconsistent evidence on predictor importance. We contribute to the state of the field by leveraging a recently constructed MULTI-PSYCH cohort (*n* = 2668) with a broad selection of potential predictors, integrating data routinely collected at the clinic with additional inputs such as PRS and a broad range of variables from Swedish population registries [45]. The aim of the present study was twofold: to identify patient characteristics predictive of higher post-treatment symptom severity and to evaluate whether incorporating genetic and register predictors alongside established clinical predictors explains additional variance in post-treatment symptom severity.

## Patients and methods

### Sample

The MULTI-PSYCH cohort study comprises 2668 adult outpatients treated with 12 weeks of ICBT for MDD (*n* = 1300), PD (*n* = 727) or SAD (*n* = 641) at the Internet psychiatry unit of the Psychiatric Clinic Southwest at Karolinska University Hospital Huddinge, Sweden, between 2008 and 2020. Screening and treatment procedures are described in more detail elsewhere [46]. As part of the assessment, patients completed an online questionnaire and were interviewed by a licensed clinician (psychologist or psychiatrist), providing sociodemographic information along with details about their condition and medical history. Blood samples were collected for DNA extraction before treatment, and data from multiple nationwide registers were linked using each patient’s unique Swedish personal number.

### Variables

Candidate predictors were divided into three groups based on their source: clinic-based (obtained as part of pre-treatment questionnaires and interviews, containing medical and sociodemographic data), genetic (PRS for seven traits derived from GWAS summary statistics and calculated for all genotyped patients), and register-based (sourced through record linkage across multiple national registers containing medical, socioeconomic, and demographic data). This division is helpful for assessing the relative contribution of predictors derived from different sources: genetic and register data are not readily available in the routine care setting, and thus, their inclusion must be justified by substantial added explanatory power.

The purpose of this study was to identify predictors that have prognostic value, and thus, selected variables must be available pre-treatment. Process variables, e.g., adherence and homework completion, were therefore excluded. To minimize predictor selection bias, clinic-based predictors were pre-selected based on literature review. To compile a list of established predictors, we reviewed studies of adults receiving CBT (internet-based or face-to-face) for MDD, PD, or SAD (see Supplementary Material, S1 Table). By contrast, register data are rarely accessible for research purposes, leading to a scarcity of literature on established predictors. Thus, primary selection of register-based variables was guided by their face validity and subsequently validated through bivariate regression analyses. Those predictors that were significantly associated with the outcome were added to the final model.

#### Clinic-based predictors

Baseline symptom severity was measured right before ICBT start with the Montgomery–Åsberg Depression Rating Scale, self-rating version (MADRS-S) for MDD (test–retest reliability: *r* =.78; internal consistency reliability: α = 0.84) [47], Panic Disorder Severity Scale, self-report (PDSS-SR) for PD (test–retest reliability: *r* =.81; internal consistency reliability: α = 0.92) [48], and Liebowitz Social Anxiety Scale, self-report (LSAS-SR) for SAD (test–retest reliability: *r* =.83; internal consistency reliability: α = 0.95) [49]. In the present study, the three disorders along with their respective predictors and outcome measures were combined to increase statistical power. While this decision comes at the cost of introducing more heterogeneity, we deemed it conceptually reasonable given the magnitude of phenotypic and etiological overlap between MDD, PD, and SAD, the similarities in the three ICBT treatment protocols, and the growing support for applying transdiagnostic models of psychopathology [50], [51], [52]. Finally, a recent machine-learning study aimed at investigating the value of pooling intervention data for MDD, PD, and SAD into a single dataset and using a larger sample derived from the same source as the current study supported the superiority of this approach [53]. The disorder-specific scales were transformed to a harmonized 0-100 range and subsequently pooled into a single score [54] (see Supplementary Material).

Comorbidity is a binary measure that indicates the presence of coexisting psychiatric disorders as assigned by a clinician during a screening interview. Family history captures self-reported confirmed or suspected cases of psychopathology among first-degree relatives selected from a list of 36 options. Sex was derived from the Swedish personal identity number and therefore refers to biological sex. Highest achieved level of education was self-reported at screening and for statistical analysis dichotomised into *University* (finished or unfinished) and *No university* (up to upper secondary school). Marital status is a self-reported civil status dichotomised into *Married* (officially married or de facto married, i.e., in a committed relationship (‘married/living together/living apart together’)) and *Unmarried (Single*, *Separated*, and *Widowed).* Finally, Parental status was retrieved from the screening question, “Do you have children?“.

#### Genetic predictors

Genotyping was performed in three batches at LIFE & BRAIN GmbH in Bonn, Germany, with Illumina HumanCoreExome-12 v1.0, Infinium Global Screening Array-24 v1.0, and Infinium Global Screening Array-24 v2.0, respectively. Quality control and imputation were performed via the RICOPILI GWAS pipeline [55]. PRS for seven traits (MDD, attention deficit hyperactivity disorder (ADHD), ASD, bipolar disorder (BPAD), schizophrenia (SCZ), IQ, and educational attainment (EA)) were constructed using the PRS-CS method. A detailed description of genetic data pre-processing can be found elsewhere [56].

#### Register-based predictors

The Swedish national population registration system provides high-quality data on important life events for all individuals who have been assigned a personal identity number and is unique in its almost complete coverage [57].

Register-based employment status was derived from the Register-based Employment Statistics (RAMS) [58] and has been dichotomised into *Employed*/*Unemployed*. Income was obtained from the Total Population Register [59] and is defined as annual disposable household income divided by consumption weight of the family, thus constituting an individual’s component of the household income adjusted for family composition. For statistical analyses, Income was divided into quintiles. Lastly, Financial benefits is receipt of any of eight benefits from the Swedish social insurance system (for a complete list, see Supplementary Material). Statistics on employment, income, and financial benefits are updated annually; hence, the most recent available record from the year preceding treatment was utilized.

Prior psychiatric diagnoses were derived from the National Patient Register (NPR) [60] and the Stockholm regional healthcare data warehouse VAL (Vårdanalysdatabasen) [61]. NPR comprises data on specialized in- and outpatient care visits in Sweden, while VAL registers all primary care visits in Stockholm County, where most of the patients reside. In addition to ICD-10 Chap. 5 codes (e.g. F3: Mood [affective] disorders), a separate variable was constructed for BPAD to avoid conflation with depressive disorders which are the target in the studied ICBT treatment. Separate variables were also created for ASD and ADHD and, given their neurodevelopmental nature and persistent lifelong manifestation, the inclusion was not limited to diagnoses received prior to ICBT treatment. To commence the ICBT treatment, a patient must receive a relevant diagnosis, either from a general practitioner or a clinician at the Internet Psychiatry clinic, which is subsequently transferred to the NPR. Hence, to avoid capturing the diagnosis related to this very healthcare episode and only focus on the *prior* events, all records of MDD/PD/SAD within a month preceding the ICBT treatment were excluded from the analysis. Data on dispensation of prescription medicines were retrieved from the National Prescribed Drug Register [62] and included Antidepressants (N06A), Anxiolytics (N05B), Hypnotics and sedatives (N05C), and Antipsychotics (N05A). As with the psychiatric diagnoses, all records of dispensation within a month preceding ICBT treatment were excluded from the analysis.

Binary variables for Education (*University*/*No university*), Marital status (*Married*/*Unmarried*), and Parental status (*Children*/*No children*) were constructed from both clinic-based and register-based data. In the full model, we chose to use clinic-based measures dictated by higher granularity of answers (e.g. meaningful partnership without marriage, etc.) and ease of data obtainment. A detailed description of this rationale and data pre-processing is provided in Supplementary Material.

#### Outcome

Treatment outcome is defined as post-treatment symptom level, measured by a self-rated, disorder-specific scale. Identically to the baseline symptom severity, harmonized scores for the three disorders were pooled.

## Statistical analysis

### Missing data

Summary of missing data is presented in Supplementary Material, S2 Table. Clinic-based predictors with over 30% missing values were excluded. When available, missing pre-treatment symptom severity values (*n* = 30, 1.1%) were replaced with those collected at screening, resulting in three missing values in the final dataset (0.1%). For all the PRS, 456 (17.1%) observations were missing due to being excluded during quality control procedures (poor matching and partial non-European ancestry). Among register-based predictors, there were six missing values in Employment, Income, and Financial benefits.

The outcome variable, post-treatment symptom severity, had 524 missing observations (19.6%). The missingness mechanism was hypothesized to be missing at random (MAR), i.e., the missingness is conditional on other variables in the dataset, and the sensitivity analysis was performed to check the robustness against departures from this assumption (see the results in Supplementary Material). The missingness pattern suggested that non-completers were more likely to have higher post-treatment symptom values since the variables associated with missingness were also predictive of higher outcome values in the non-missing subset. Thus, listwise deletion (complete-case analysis) was ruled out for two reasons: it negatively affects the precision of estimates by decreasing power and, more importantly, if missing data are not MCAR, it is likely to introduce selection bias into inferences and cause too conservative estimates [63], [64], [65], [66], [67], [68], [69].

To address the uncertainty introduced by missingness, missing values were imputed with multiple imputation using the R package *mice *[70]. A total of *m* = 20 datasets were imputed over 20 iterations. In accordance with the recommended procedure, 25 auxiliary variables not included in the analytical sample were added to the imputation dataset to improve the quality of imputations through increasing the plausibility of the MAR assumption [66]. Missing values were imputed using the default settings of the *mice* package. For numeric data, predictive mean matching (*pmm*) was used as the imputation method, replacing a missing value with a randomly selected observed donor that has a regression-predicted value closest to the missing value based on a simulated regression model. It is a superior method to regression in that it does not rely on the assumption of joint multivariate normal distribution and creates realistic imputed values. For binary data, logistic regression imputation (*logreg*) was used and for unordered categorical data, polytomous regression imputation (*polyreg*). To check the adequacy of the imputation models and assess convergence, both graphic and numeric diagnostic methods were employed, namely kernel density plots of the distributions of the observed and *m* = 20 imputed datasets and the Kolmogorov-Smirnov test to assess departures from the assumptions made in the imputation model [68]. In addition, we performed a complete-case sensitivity analysis for the full multiple regression model.

### Main analysis

All statistical analyses were performed using R (version 4.3.1). First, univariate analyses were performed on all predictors to examine their distribution and properties. Next, two models were developed: a baseline model (clinic-based data only) and a full model (clinic-based, genetic, and register data). The same steps of statistical analysis were repeated for both models: first, separate bivariate linear regression models were fit to each predictor; next, all covariates were entered simultaneously into the multiple linear regression model. The purpose of conducting the analysis in two steps was dictated by the research questions. Given the complex nature of the studied phenomenon and the known associations between the covariates from existing literature on latent human traits, at least partial collinearity is inevitable. Thus, to evaluate independent associations between individual predictors and the outcome, assessment of simple regression coefficients is necessary to avoid misinterpreting small effect sizes, whereby a variable important in a bivariate model might be attenuated in a multiple model by other collinear predictors. For the second goal of the overall model appraisal, the accuracy of point estimates is not paramount. Therefore, variable selection was theory-driven, and all pre-selected variables were retained in the multiple regression model irrespective of their statistical significance. This was done to avoid the flaws related to the algorithm of stepwise variable selection whereby variables are added and removed based on their significance level [71].

A total of 45 predictors were used in the full model. All variables with zero variance were excluded from the analysis. Variables with very low variance (meeting both criteria: frequency of the second most common value of < 1% over the sample and percentage of unique values of < 10% of the total number of data points) (*n* = 46) were excluded as independent predictors but retained within relevant composite variables. For a full list of included and excluded variables, see Supplementary Material. For prior diagnoses and medication, both composite and individual components were assessed through bivariate analyses for the sake of potentially discovering novel predictors. For example, using a composite variable that encompasses any prior psychotropic medication may be justified in the interest of model parsimony, but it may miss what specific medication drives the effect size of the association. However, only individual predictors were used in multiple regression analysis to avoid multicollinearity. Owing to the exploratory nature of this study and the underlying goal to potentially discover novel predictors, in the main analysis, we did not apply correction for multiple comparisons due to the risk of introducing type II error [72]. However, we performed a post-hoc false discovery rate (FDR) control for the multiple regression model using the Benjamini–Hochberg (BH) procedure.

Lastly, the baseline and the full model were evaluated based on their goodness of fit using adjusted coefficients of determination ($\:{R}_{adj}^{2}$). All additional covariates are likely to increase $\:{R}^{2}$, regardless of their true explanatory power. In contrast, $\:{R}_{adj}^{2}$ imposes a penalty for this inflation when comparing models with a varying number of predictors. Consequently, the assessment of the full model performance compared to the baseline model relied on the extra variance explained by additional predictors and was complemented with the appraisal of the Akaike information criterion (AIC) and root-mean-square error (RMSE) of the two models.

## Results

### Descriptive statistics

Sample characteristics are presented in Table 1 (for more details, see Supplementary Material, S4 Table). Descriptive statistics for the observed, imputed, and complete dataset, as well as the distributional discrepancy between observed and imputed outcome values, are provided in Supplementary Material, S5 Table and Supplementary Material, Figure S1, respectively.

**Table 1:** Descriptive statistics for the total sample and stratified by disorder

Variable	Total (*n* = 2668)	MDD (*n* = 1300)	PD (*n* = 727)	SAD (*n* = 641)
Pre-treatment symptom severity, mean (SD)	47.6 (17.8)	51.6 (15.7)	40.2 (18.8)	47.7 (17.9)
Post-treatment symptom severity, mean (SD)	28.2 (18.8)	30.3 (18.7)	17.8 (16.1)	35.3 (17.2)
Relative symptom change, % (SD)	-41.3 (37.9)	-41.7 (34.3)	-53.4 (47.0)	-28.1 (26.5)
Age, mean (SD)	35.6 (11.4)	37.6 (11.9)	34.6 (10.8)	32.7 (10.3)
Male sex, *n* (%)	1014 (38.0%)	443 (34.1%)	292 (40.2%)	279 (43.6%)
Psychiatric comorbidities, *n* (%)	862 (33.5%)	394 (31.4%)	265 (37.9%)	203 (32.9%)
Family history of psychopathology, *n* (%)	1812 (70.6%)	888 (71%)	488 (69.7%)	436 (70.7%)
Self-reported married, *n* (%)	1566 (58.8%)	727 (56.1%)	477 (65.7%)	362 (56.7%)
Self-reported having children, *n* (%)	1102 (41.4%)	603 (46.4%)	302 (41.5%)	199 (31.0%)
Self-reported university education, *n* (%)	1693 (63.6%)	890 (68.8%)	406 (55.9%)	397 (62.1%)
Register-based employed, *n* (%)	2448 (92.0%)	1205 (92.7%)	674 (92.8%)	571 (89.1%)
Annual income (SEK), mean (SD)	241,713 (201,190)	256,187 (202,155)	244,258 (232,968)	209,591 (150,742)
Financial benefits in the past year, *n* (%)	556 (20.9%)	302 (23.3%)	148 (20.4%)	106 (16.5%)
Prior psychiatric diagnosis, *n* (%)	1793 (67.2%)	895 (68.8%)	518 (71.3%)	380 (59.3%)
Prior psychotropic medication, *n* (%)	1665 (62.4%)	863 (66.4%)	472 (64.9%)	330 (51.5%)

SD, standard deviation; SEK, Swedish krona; MDD, Major depressive disorder; PD, Panic disorder; SAD, Social anxiety disorder

### Predictor importance

To assess potential differences in predictor importance across disorders, we tested for the effect modification by diagnosis (MDD, PD, or SAD) by including an interaction term for each predictor and then applying a Wald test to assess whether any interactions collectively differ from zero. The results suggested no evidence of significant moderation of predictor effects by disorder (global test of all interaction terms: F(88, 1595) = 1.15, *p* =.172).

Parameter estimates for clinic-based, genetic, and register-based predictors are displayed in Table 2.

**Table 2:** Association between clinic-based, genetic, and register-based predictors and treatment outcome. Results from bivariate and multiple regression analyses in the full model

Predictor	Bivariate		Multiple	
	Estimate (95% CI)	*p*-value	Estimate (95% CI)	*p*-value
Pre-treatment score	0.57 (0.53, 0.61)	< 0.001***	0.51 (0.47, 0.55)	< 0.001***
Comorbidities	5.20 (3.54, 6.86)	< 0.001***	0.42 (-1.03, 1.88)	0.569
Family history	2.75 (0.92, 4.58)	0.003**	0.19 (-1.41, 1.79)	0.816
Sex (ref: male)	0.58 (-1.06, 2.23)	0.488	-2.56 (-4.03, -1.09)	< 0.001***
Unmarried	4.62 (2.89, 6.34)	< 0.001***	1.67 (0.08, 3.26)	0.040*
No children	2.28 (0.72, 3.85)	0.004**	0.72 (-0.84, 2.28)	0.364
No university	4.18 (2.52, 5.85)	< 0.001***	1.95 (0.49, 3.42)	0.009**
Unemployed	7.45 (4.15, 10.75)	< 0.001***	2.98 (-0.01, 5.97)	0.051
Income (quintiles)	-1.90 (-2.47, -1.34)	< 0.001***	-0.81 (-1.38, -0.25)	0.005**
Any financial benefits	6.24 (4.23, 8.25)	< 0.001***	2.24 (0.37, 4.11)	0.019*
PRS MDD	0.71 (-0.19, 1.60)	0.120	0.41 (-0.39, 1.21)	0.310
PRS ASD	0.58 (-0.37, 1.53)	0.228	0.43 (-0.45, 1.32)	0.335
PRS ADHD	0.68 (-0.33, 1.69)	0.182	0.14 (-0.71, 0.99)	0.741
PRS BPAD	-0.21 (-1.07, 0.66)	0.640	-0.73 (-1.57, 0.12)	0.091
PRS Education	-0.30 (-1.24, 0.64)	0.527	0.24 (-0.73, 1.20)	0.630
PRS IQ	0.37 (-0.59, 1.33)	0.444	0.38 (-0.58, 1.34)	0.434
PRS SCZ	0.28 (-0.63, 1.19)	0.544	0.46 (-0.45, 1.38)	0.315
Any prior psychiatric diagnosis	5.54 (3.88, 7.20)	< 0.001***		
Prior F1	3.16 (-0.07, 6.39)	0.055	-1.76 (-4.63, 1.11)	0.230
Prior F3 (excl. BPAD)	7.61 (5.89, 9.34)	< 0.001***	2.29 (​​0.56, 4.02)	0.010*
Prior F4	3.29 (1.69, 4.90)	< 0.001***	0.93 (-0.61, 2.48)	0.235
Prior F5	7.84 (5.05, 10.64)	< 0.001***	2.70 (0.11, 5.30)	0.041*
Prior F6	15.70 (9.35, 22.06)	< 0.001***	5.55 (-0.11, 11.20)	0.055
ASD	24.98 (18.35, 31.61)	< 0.001***	10.35 (4.17, 16.53)	0.001**
ADHD	15.98 (12.24, 19.71)	< 0.001***	6.38 (2.91, 9.85)	< 0.001***
Any prior medication	4.52 (2.89, 6.16)	< 0.001***		
Antidepressants	6.30 (4.66, 7.94)	< 0.001***		
Bupropion	14.16 (9.75, 18.58)	< 0.001***	6.04 (1.85, 10.24)	0.005**
Duloxetine	10.84 (6.07, 15.61)	< 0.001***	-0.49 (-4.87, 3.90)	0.827
Fluoxetine	10.52 (6.97, 14.07)	<0.001***	4.18 (0.91, 7.44)	0.012*
Venlafaxine	9.44 (5.77, 13.10)	< 0.001***	2.78 (-0.62, 6.18)	0.108
Mirtazapine	8.31 (5.12, 11.50)	< 0.001***	0.48 (-2.62, 3.59)	0.759
Amitriptyline	7.93 (2.89, 12.96)	0.002**	4.04 (-0.27, 8.35)	0.066
Sertraline	6.51 (4.56, 8.47)	< 0.001***	2.70 (0.88, 4.52)	0.004**
Escitalopram	6.42 (3.63, 9.21)	< 0.001***	1.77 (-0.72, 4.27)	0.163
Citalopram	3.74 (1.60, 5.87)	< 0.001***	2.20 (0.22, 4.18)	0.030*
Clomipramine	1.71 (-5.96, 9.38)	0.662	-2.16 (-8.73, 4.41)	0.518
Paroxetine	-1.49 (-5.97, 2.98)	0.512	-0.09 (-3.95, 3.77)	0.963
Anxiolytics	3.14 (1.40, 4.89)	< 0.001***		
Buspirone	19.41 (11.63, 27.20)	< 0.001***	7.73 (0.61, 14.84)	0.033*
Alprazolam	4.00 (-1.60, 9.59)	0.161	1.70 (-3.23, 6.62)	0.498
Hydroxyzine	3.16 (1.26, 5.05)	0.001**	-1.78 (-3.72, 0.15)	0.070
Diazepam	2.59 (-1.36, 6.55)	0.198	-1.15 (-4.60, 2.29)	0.512
Oxazepam	2.25 (-0.02, 4.53)	0.052	-0.84 (-3.00, 1.32)	0.444
Hypnotics and sedatives	6.20 (4.40, 7.99)	< 0.001***		
Melatonin	11.02 (5.74, 16.30)	< 0.001***	2.56 (-2.31, 7.42)	0.301
Zolpidem	6.23 (3.46, 9.01)	< 0.001***	0.91 (-1.73, 3.56)	0.498
Zopiclone	5.79 (3.40, 8.18)	< 0.001***	0.03 (-2.49, 2.43)	0.984
Propiomazine	5.18 (2.80, 7.56)	< 0.001***	-1.56 (-3.91, 0.79)	0.193
Antipsychotics	5.98 (1.37, 10.59)	0.011*	-3.63 (-7.79, 0.53)	0.087

PRS, polygenic risk score; MDD, Major depressive disorder; ASD, Autism spectrum disorder; ADHD, Attention deficit hyperactivity disorder; BPAD, Bipolar affective disorder; IQ, intelligence quotient; SCZ, Schizophrenia; F1, Mental and behavioural disorders due to psychoactive substance use; F3, Mood [affective] disorders; F4, Anxiety, dissociative, stress-related, somatoform and other nonpsychotic mental disorders; F5, Behavioural syndromes associated with physiological disturbances and physical factors; F6,Disorders of adult personality and behaviour; CI, confidence interval

Significance levels: *p <.05, **p <.01, ***p <.001

As expected, pre-treatment symptom level was a strong predictor of post-treatment score severity (β = 0.57, 95% CI [0.53, 0.61], *p* <.001), remaining almost unattenuated in the adjusted model. No significant association between PRS and the outcome was observed. Socioeconomic predictors (no university education, unemployment, receipt of financial benefits, and lower income quintile) were strongly associated with higher post-treatment symptom severity and remained significant in the multiple regression model. Being childless was associated with a higher post-treatment score in the bivariate model, but the effect disappeared in the adjusted model. By contrast, female sex showed association with lower post-treatment score in the adjusted model only.

Having a history of psychiatric diagnoses predicted higher post-treatment symptom severity. In addition to previous records of depressive and anxiety disorders, eating and personality disorders were strongly associated with poorer treatment outcome. A particularly large effect sizes were found for comorbid ASD (β = 24.98, 95% CI [18.35, 31.61], *p* <.001) and ADHD (β = 15.98, 95% CI [12.24, 19.71], *p* <.001), the observed relationship persisted in the adjusted model. However, due to relatively small sizes of ASD (*n* = 41) and ADHD samples (*n* = 133), these results need to be interpreted with caution.

The other significant predictor was prior use of almost all psychotropic medications. In addition to the substantial effect of most antidepressants and some anxiolytics (particularly buspirone), it is worth emphasizing quite a strong association with the prior use of hypnotics and sedatives (with a notably large effect of melatonin) as well as antipsychotics.

Results of the complete-case sensitivity analysis suggest that the pattern and magnitude of associations in the complete-case dataset is largely similar to that of the imputed dataset (see Supplementary Material, S6 Table).

The BH correction at FDR = 5% applied to the multiple regression model yielded the largest passing rank of *i* = 9 and a *p*-value threshold of 0.010. The following seven predictors passed their rank thresholds and remained significant: pre-treatment symptom level, sex, income, comorbid ASD, comorbid ADHD, prior use of bupropion, and prior use of sertraline.

We also conducted a post-hoc exploratory analysis with symptom change as the outcome, defined as the percentage difference between pre- and post-treatment scores. The results of multiple regression are presented in Supplementary Material, S7 Table. Of note, predictors of symptom change differ from those of post-treatment symptom severity, indicating that the two traits are very different. Specifically, baseline severity, the strongest predictor of post-treatment score, is not significant for symptom change. In contrast, marital status, receipt of financial benefits, comorbid ASD and ADHD diagnoses, and prior use of some psychotropic medications are predictive for symptom change, too.

### Model performance

**Fig. 1:**
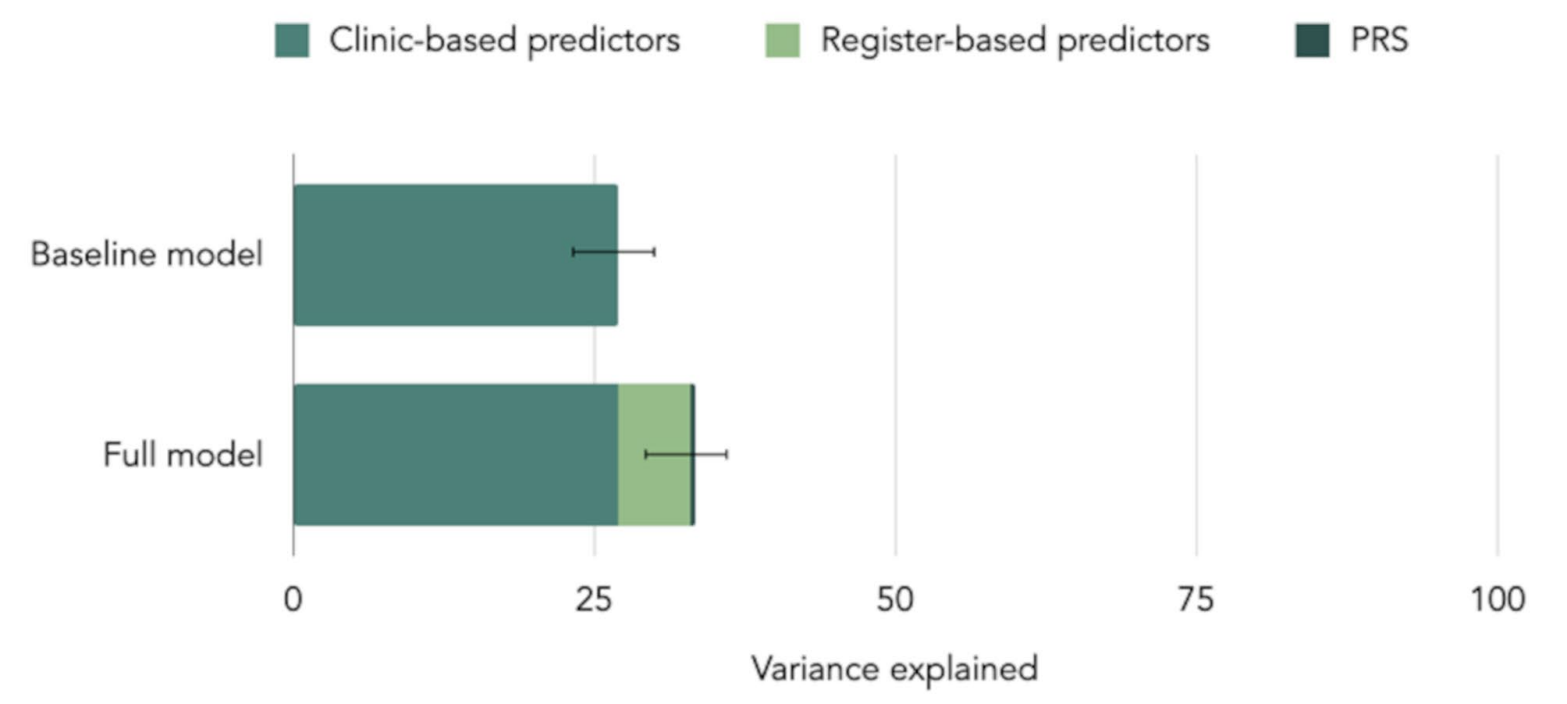
Compared variance explained by baseline and full model by predictor type

Estimates of comparative performance of the models are presented in Table 3 and visualized in Fig. 1. $\:{R}_{adj}^{2}$ of the baseline model was 0.27, thus explaining 27% of the variance in post-treatment symptom severity, which is almost entirely driven by pre-treatment symptom severity. The full model yielded $\:{R}_{adj}^{2}$ of 0.34. Addition of PRS did not contribute to the model performance, increasing $\:{R}_{adj}^{2}$ of the full model by only 0.2% points above clinic-based and register-based predictors. The full model was also superior when comparing the two models’ AIC and RMSE. These results suggest that including rich register data provided some additional explanatory power beyond the clinic-based predictors used in the baseline model, accounting for a further 7% of the variance.

**Table 3:** Adjusted R-squared ($\:{R}_{adj}^{2}$), Akaike information criterion (ΔAIC), and root mean square error (RMSE) values of baseline and full models

Model	$\:{R}_{adj}^{2}$	ΔAIC	RMSE	# of predictors
Baseline model	0.27	22613.5	15.2	6
Full model	0.34	22411.2	14.3	45

$\:{R}_{adj}^{2}$, Adjusted R-squared; ΔAIC, Akaike information criterion; RMSE, Root mean square error

## Discussion

The first goal of this study was to identify predictors of post-treatment symptom severity in patients with mild to moderate MDD, PD, and SAD treated with highly standardized ICBT protocols and using a large and diverse pool of clinical, genetic, and register predictors. Our findings support the previously established importance of pre-treatment symptom severity, psychiatric comorbidities, family history of psychopathology, and socioeconomic status. In line with existing research on the link between marital status and mental health, being single, divorced or widowed was predictive of a poorer treatment outcome. Furthermore, thanks to unique data sources available in this study, we were able to assess a variety of predictors that have not been investigated before: the role of prior and concurrent psychiatric diagnoses and specific medications is a novel addition of this study, as are socioeconomic predictors such as income and receipt of financial benefits.

Identification of hindering factors associated with poor treatment outcomes bears potential clinical relevance. Patients with a longer history of psychiatric morbidity, particularly prior depression, anxiety, eating, and personality disorders, as well as those with the history of psychotropic medication use, represent a vulnerable clinical sub-population and require more attention to prevent high residual symptomatology after treatment. While these characteristics appear to be associated with higher burden of psychopathology, controlling for baseline severity did not fully attenuate their effect, possibly suggesting a different patient profile. Screening for these factors may help clinicians direct such patients to more appropriate higher-intensity interventions as well as potentially consider integrated treatment formats targeting existing comorbidities. Addressing comorbid ASD and ADHD appears particularly urgent based on our findings. The need to adjust psychotherapeutic interventions for depression and anxiety to the patient’s ASD symptomatology has long been acknowledged. Socio-communication impairment, difficulties with introspection, and limited cognitive flexibility have been suggested as impeding treatment effectiveness [73]. Consequently, multiple CBT adaptations for patients with comorbid ASD have been developed [74]. The discovery that in this patient group, the average post-treatment score was nearly twice as high as for patients without ASD potentially highlights absence of relevant modifications in the studied ICBT treatment. In patients with comorbid ADHD, the findings may reflect a similar lack of treatment accommodation. Perhaps unsurprisingly, maintaining attention and organizing oneself over a 12-week treatment period, which involves extensive homework and the absence of immediate therapist guidance that is characteristic of ICBT, may pose a challenge for this patient group and calls for adaptive treatment strategies to avoid treatment failure [75].

Our results also suggest a substantial contribution of lower socioeconomic status to poorer treatment success. Background factors of patients treated with ICBT receive less attention compared to traditional in- and outpatient facilities. Our findings, however, underscore the importance of addressing them through e.g. early involvement of social services to mitigate adverse outcomes.

The application of PRS in psychiatry is promising as it is a constant throughout lifetime and can be used for long-term prediction. Yet, their limitation is that on a population level, PRS tend to follow a Gaussian distribution with significant overlap between cases and controls, and they only explain a small fraction of genetic variation, which in turn explains around 50% of phenotypic variation. Failure to detect the association between PRS and post-treatment symptom severity in our study is perhaps not surprising. We used PRS related to psychopathology and personality, and, while genetic liability to these traits is likely to be shared with that of treatment outcome, it does not necessarily follow that the same PRS will be helpful in predicting it. Thus, a specific PRS constructed from GWAS of treatment response, which is still missing, could potentially yield a stronger effect.

The second goal of the study was to assess whether employing a wider range of predictors would be advantageous in identifying patients who will have higher post-treatment symptom severity. We found that the full model with multimodal predictors had a superior performance in explaining the variance in post-treatment severity compared to the baseline model. However, the improvement in explained variance was modest. One potential interpretation is that even though the evaluated predictors encompass a distinct range of properties, they might, to a certain degree, be considered as capturing a shared underlying construct and providing little independent information. For example, genetic predictors will inevitably be correlated due to vertical pleiotropy, whereby the same genetic variant affects multiple different traits, e.g., a relevant SNP influences intelligence, which serves as a mediator influencing educational attainment, SES, and, in turn, post-treatment symptom severity. While the collinearity of independent variables does not negatively affect the explanatory power of the whole model, it does not improve it either, since many variables explain an overlapping proportion of variance in the outcome. Further, the full model would likely benefit from the inclusion of additional strong predictors. For example, in the exploratory complete-case analysis, duration of symptoms was strongly associated with the outcome in its effect size and variance explained but could not be included as it surpassed the predefined threshold for the allowed amount of missingness. Moreover, human complex traits are dominated by stochasticity and are often attributed to idiosyncratic factors which remain largely unmeasured. Another possibility is that pooling the three disorders makes the studied phenotype more heterogeneous and possibly dilutes the findings. Also, while the current sample size exceeds those in the earlier studies, it may still not be sufficiently powered. Finally, the relationship between predictors and the outcome may be non-linear and contain underlying interaction effects, making classical ordinary least squares modelling unsuitable.

### Strengths and limitations

The main strength of this study is its relatively large sample size and diversity of evaluated predictors. Moreover, register-based data have almost no missing values and are highly reliable. Another strength is a homogenous group of patients that completed a highly protocolized treatment, which allows for meaningful comparisons of the outcome. Deriving the data from routine care has additional benefits since most studies of treatment outcome constitute a primary or secondary analysis of RCT data, where user characteristics are subject to stringent inclusion criteria, thus introducing selection bias and limiting the generalisability [76]. However, a potential limitation in the applicability of the findings to other populations needs to be mentioned. All the study participants lived in Sweden, were more educated than the general population, were fluent in Swedish, and exhibited mild to moderate symptom severity. In addition, there is a significant self-selection bias, with most patients self-referring to the Internet psychiatry clinic.

Finally, it must be emphasized that the findings of this study cannot be viewed as supporting the causal nature of the relationship between any of the predictors and the outcome. While the terms *treatment outcome* and *treatment response* do semantically assume, at least partially, that the post-treatment symptom measure is conditional upon and a direct consequence of the intervention, no such claims can be made given the non-experimental design of the study. However, causal language is widely and loosely applied across the literature and, in the interest of brevity and recognition, is also sometimes used in this paper, more so when referring to the previous findings rather than the current study, where we try to adapt the more observational term *post-treatment symptom severity*. This operationalization of the response variable was chosen as it is deemed by the authors as the most appropriate measure to be predicted at the baseline. Since the goal of the future predictive model is not to assess treatment effectiveness, relative symptom change was not of interest here. Moreover, even when the percentage change is substantial and passes some predefined threshold, the patient may still be symptomatic beyond what is intended. Choosing remission as the outcome was rejected due to its somewhat arbitrary cut-off, loss of information, and diminished statistical power that ensues from dichotomization.

## Conclusions

The current study adds to the growing literature on predictors of treatment outcome by supporting established ones and suggesting some novel predictors. It also proposes that a statistical model based on a few relatively easily obtainable measures explains a comparable proportion of the variance in the outcome as a model containing a much broader array of multimodal data. As the next step, a machine learning model should be developed to address non-linear associations and higher-order interactions between input features to perform predictions at individual patient level. Additional future endeavours may include leveraging all available SNP information beyond what meets a strict GWAS significance threshold and appraising an even larger and more diverse pool of register-based predictors.

## Electronic supplementary material

Below is the link to the electronic supplementary material.

Supplementary Material 1

## Data Availability

The dataset analysed during the current study is not publicly available due to the sensitive nature of the data.
