# Supplementary material for: Clinical, genetic, and sociodemographic predictors of symptom severity after internet-delivered cognitive behavioural therapy for depression and anxiety
Source: BMC Psychiatry. 2025 May 30;25:555. doi: 10.1186/s12888-025-07012-x (PMC12125921; doi:10.1186/s12888-025-07012-x)
Supplement: Supplementary file 1 — Supplementary Material 1 [file 12888_2025_7012_MOESM1_ESM.docx]

**Supplementary Material for**

**Clinical, genetic, and sociodemographic predictors of symptom severity after internet-delivered cognitive behavioral therapy for depression and anxiety**

**Olly Kravchenko^1^, Julia Bäckman^1^, David Mataix-Cols^1^, James Crowley^1,2^, Matthew Halvorsen^1,2^, John Wallert*^1^, Christian Rück*^1^**

*Shared last author

^1^Centre for Psychiatry Research, Department of Clinical Neuroscience, Karolinska Institutet, & Stockholm Health Care Services, Region Stockholm, Sweden

^2^Department of Genetics, University of North Carolina at Chapel Hill, Chapel Hill, USA

TABLE OF CONTENTS

S1 Table. Established predictors of symptom change following internet-delivered or traditional CBT treatment of MDD, PD, and SAD 2

Harmonization of three disorder-specific scales 5

Description of financial benefits 6

Variable description and preprocessing 7

S2 Table. Summary of missing data for variables in the analytic sample 10

S3 Table. Full list of predictors used in baseline and full models by source of data 11

Variables with near-zero variance excluded from the analysis 14

Sensitivity analysis of outcome missingness 16

S4 Table. Detailed descriptive statistics for all disorders 17

S5 Table. Descriptive statistics for the observed, imputed, and complete data 19

S6 Table. Results of multiple regression on a complete case dataset 20

S7 Table. Results of multiple regression with symptom change as the outcome 22

Figure S1. Density plot of observed and imputed outcome data 24

**S1 Table.** Established predictors of symptom change following internet-delivered or traditional CBT treatment of MDD, PD, and SAD

| **Predictor** | **Direction of association with symptom reduction** | **Comment** | **Diagnosis** | **Reference** |
| --- | --- | --- | --- | --- |
| Pre-treatment symptom severity | Positive |  | SAD | [El Alaoui et al. (2015](https://doi.org/10.1136/bmjopen-2015-007902))  PMID: 26105031 |
|  |  |  | MDD, anxiety disorders | [Edmonds et al. (2018](https://doi.org/10.1016/j.janxdis.2018.01.003))  PMID: 29421369 |
|  |  |  | MDD | [Button et al. (2012)](https://doi.org/10.1007/s00127-011-0389-1)  PMID: 21541696 |
|  |  |  | MDD, GAD | [Hadjistavropoulos et al. (2016)](https://doi.org/10.1016/j.beth.2015.10.006)  PMID: 26956649 |
|  | Negative |  | SAD | [Nordgreen et al. (2012)](https://doi.org/10.1016/j.brat.2011.10.009)  PMID: 22134140 |
|  |  |  | MDD | [Andersson et al. (2004)](https://doi.org/10.1159/000080914)  doi.org/10.1159/000080914 |
|  |  |  | MDD | [Hamilton & Dobson (2002)](https://doi.org/10.1016/s0272-7358(02)00106-x)  PMID: 12214329 |
|  |  |  | MDD | [Jarrett et al. (1991)](https://doi.org/10.1016/0165-1781(91)90061-s)  PMID: 1891508 |
|  |  |  | PD | [Black et al. (1994)](https://doi.org/10.1016/0165-0327(94)90131-7)  PMID: 8014323 |
|  |  |  | Multiple | [Rozental et al. (2019)](https://doi.org/10.3389/fpsyg.2019.00589)  PMID: 30984061 |
|  |  |  | MDD | [De Graaf et al. (2010)](https://doi.org/10.1037/a0018324)  PMID: 20350029 |
|  |  |  | MDD | [Wallert et al. (2022)](https://www-nature-com.proxy.kib.ki.se/articles/s41398-022-02133-3)  PMID: 36050305 |
| Comorbidity with mood/anxiety disorders | Negative | Anxiety | SAD | [Mululo et al. (2012)](https://doi.org/10.1590/s1516-44462012000100016)  PMID: 22392395 |
|  |  |  | SAD | [Hedman et al. (2012)](https://doi.org/10.1111/j.1600-0447.2012.01834.x)  PMID: 22320999 |
|  |  |  | Multiple | [Rozental et al. (2019)](https://doi.org/10.3389/fpsyg.2019.00589)  PMID: 30984061 |
|  |  | Depression | SAD | [Chambless et al. (1997)](https://doi.org/10.1016/s0887-6185(97)00008-x)  PMID: 9220298 |
|  |  |  | SAD | [Hedman et al. (2012)](https://doi.org/10.1111/j.1600-0447.2012.01834.x)  PMID: 22320999 |
|  |  |  | SAD | [Scholing & Emmelkamp (1999)](https://doi.org/10.1016/s0005-7967(98)00175-2)  PMID: 10402691 |
| Comorbidity with personality disorders | Negative |  | SAD | [Chambless et al. (1997)](https://doi.org/10.1016/s0887-6185(97)00008-x)  PMID: 9220298 |
|  |  |  | PD | [Black et al. (1994)](https://doi.org/10.1016/0165-0327(94)90131-7)  PMID: 8014323 |
|  |  |  | Anxiety disorders | [Ociskova et al. (2016)](https://doi.org/10.2147/NDT.S104301)  PMID: 27445474 |
|  |  |  | SAD | [Mululo et al. (2012)](https://doi.org/10.1590/s1516-44462012000100016)  PMID: 22392395 |
| Longer symptom duration | Negative |  | SAD | [Mululo et al. (2012)](https://doi.org/10.1590/s1516-44462012000100016)  PMID: 22392395 |
|  |  |  | MDD | [Hamilton & Dobson (2002)](https://doi.org/10.1016/s0272-7358(02)00106-x)  PMID: 12214329 |
|  |  |  | PD | [Kolek et al. (2019)](https://pubmed.ncbi.nlm.nih.gov/32112548/)  PMID: 32112548 |
|  |  |  | SAD | [Kamaradova et al. (2014)](http://www.rediviva.sav.sk/56i3/91.pdf) |
|  |  |  | Multiple | [Salomonsson et al. (2020)](https://www.tandfonline.com/doi/full/10.1080/16506073.2019.1669701)  PMID: 31638472 |
| Family history | Negative |  | SAD | [El Alaoui et al. (2015)](https://doi.org/10.1136/bmjopen-2015-007902)  PMID: 26105031 |
| Employment | Positive |  | SAD | [Hedman et al. (2012)](https://doi.org/10.1111/j.1600-0447.2012.01834.x)  PMID: 22320999 |
|  |  |  | MDD | [El Alaoui et al. (2016)](https://doi.org/10.1371/journal.pone.0161191)  PMID: 27618548 |
|  |  |  | PD | [Kolek et al. (2019)](https://pubmed.ncbi.nlm.nih.gov/32112548/)  PMID: 32112548 |
|  |  |  | Anxiety disorders | [Schat et al. (2013)](https://doi.org/10.1016/j.jpsychires.2013.09.005)  PMID: 24074517 |
|  |  |  | MDD | [De Graaf et al. (2010)](https://doi.org/10.1037/a0018324)  PMID: 20350029 |
|  |  |  | MDD | [Falconnier (2009)](https://pubmed.ncbi.nlm.nih.gov/19485632/)  PMID: 19485632 |
| Education | Positive |  | Multiple | [Salomonsson et al. (2020)](https://www.tandfonline.com/doi/full/10.1080/16506073.2019.1669701)  PMID: 31638472 |
|  |  |  | MDD | [Wallert et al. (2022)](https://www-nature-com.proxy.kib.ki.se/articles/s41398-022-02133-3)  PMID: 36050305 |
|  | Negative |  | MDD, anxiety disorders | [Edmonds et al. (2018)](https://doi.org/10.1016/j.janxdis.2018.01.003)  PMID: 29421369 |
| Marital status | Positive |  | MDD | [Jarrett et al. (1991)](https://doi.org/10.1016/0165-1781(91)90061-s)  PMID: 1891508 |
|  | Negative |  | MDD | [Button et al. (2012)](https://doi.org/10.1007/s00127-011-0389-1)  PMID: 21541696 |
| Sex | Positive | Female | Multiple | [Rozental et al. (2019)](https://doi.org/10.3389/fpsyg.2019.00589)  PMID: 30984061 |
| Adherence | Positive |  | SAD | [El Alaoui et al. (2015)](https://doi.org/10.1371/journal.pone.0124258)  PMID: 26105031 |
|  |  |  | SAD | [Hedman et al. (2012)](https://doi.org/10.1111/j.1600-0447.2012.01834.x)  PMID: 22320999 |
|  |  |  | Multiple | [Salomonsson et al. (2020)](https://www.tandfonline.com/doi/full/10.1080/16506073.2019.1669701)  PMID: 31638472 |
|  |  |  | MDD | [El Alaoui et al. (2016)](https://doi.org/10.1371/journal.pone.0161191)  PMID: 27618548 |
| Expectancy | Positive |  | SAD | [Chambless et al. (1997)](https://doi.org/10.1016/s0887-6185(97)00008-x)  PMID: 9220298 |
|  |  |  | MDD | [Beard et al. (2016)](https://pubmed.ncbi.nlm.nih.gov/26934333/)  PMID: 26934333 |
| Homework | Positive |  | Multiple | [Mausbach et al. (2010)](https://doi.org/10.1007/s10608-010-9297-z)  PMID: 20930925 |
| Alliance | Positive |  | MDD | [Bur et al. (2022)](https://doi.org/10.1016/j.invent.2022.100593)  PMID: 36471704 |
| Disability | Positive |  | MDD, anxiety disorders | [Edmonds et al. (2018)](https://doi.org/10.1016/j.janxdis.2018.01.003)  PMID: 29421369 |

**Harmonization of three disorder-specific scales**

Pre- and post-treatment symptom severity was measured with Montgomery–Åsberg Depression Rating Scale, self-rating version (MADRS-S) for MDD, Panic Disorder Severity Scale, self-report (PDSS-SR) for PD, and Liebowitz Social Anxiety Scale, self-report (LSAS-SR) for SAD.

The harmonized pre- and post-treatment score was computed based on the theoretical range of the respective scale and transformed to the range 0-100.

Example for MDD:

$x_{harmonized}$ *=* $\frac{(x - x_{min})}{{(x}_{max} - x_{min})}$ ** 100*

where $x_{min}$ is 0 and $x_{max}$ is 54

Since every patient filled in only a psychometric scale for their individual primary diagnosis, each of the 3 variables (harmonized MDD score, harmonized PD score, and harmonized SAD score) contains NA in two out of three scores irrelevant for the specific patient (i.e. each patient has exactly one non-missing disorder-specific measure). The harmonized scores for each disorder were thus unified into a composite “harmonized score” variable with the *coalesce*() function.

**Description of financial benefits:**

1. Sickness/work injury benefit (sjukpenning/arbetsskadesjukpenning), paid out by the Swedish Social Insurance Agency in case of prolonged sickness (over 2 weeks)
2. Sickness compensation (sjukersättning)/activity compensation (aktivitetsersättning), previously referred to as early retirement, is compensation for individuals who are prevented from working full-time ever in the future due to serious sickness, most commonly a psychiatric disorder
3. Rehabilitation compensation (rehabiliteringsersättning), paid out during the sickness if an individual undergoes vocational training
4. Unemployment benefit (arbetslöshetsersättning), applies to individuals who are currently unemployed but have worked previously
5. Compensation for participation in active labor market policies (ALMPs)
6. Compensation for the individuals who are partaking in subsidized employment with the Swedish Public Employment Service (åtgärdssysselsättning)
7. Social assistance (ekonomiskt bidrag), provided to households that experience economic hardships and require support to pay fixed expenses
8. Child care allowance (vårdbidrag) is financial assistance to parents with children who have special needs

**Variable description and preprocessing**

Comorbid diagnoses can be received during the screening interview and are selected from a list of 14 options (PD, SAD, mild, moderate or severe MDD, recurrent mild, moderate or severe MDD in remission, recurrent MDD with no current symptoms, agoraphobia, dysthymia, general anxiety disorder, irritable bowel syndrome (IBS), IBS without diarrhea) or filled in manually (3 fill-in fields).

**Clinic-based vs register-based sociodemographic variables**

**Education**

Clinic-based (self-reported at screening) education is the highest achieved education, divided into categories: *<7 years*, *7-9 years*, *Unfinished gymnasium*, *Vocational school*, *Gymnasium*, *Unfinished university*, and *Finished university*. For statistical analysis, dichotomised into *University* (*Unfinished university* and *Finished university*) and *No university* (*<7 years*, *7-9 years*, *Unfinished gymnasium*, *Vocational school*, *Gymnasium*).

Register-based education is highest-achieved education within one year preceding the treatment. Dichotomized into *University* (*Upper secondary education of more than 2 years* and *Postgraduate education*) and *No university* (*< 9 years*, *9 years*, *Gymnasium* and *Upper secondary education of less than 2 years*).

Sizes of the self-reported and register-based Education sub-categories vary slightly due to somewhat differing classifications (self-reported *University* (*n* = 1693) and *No university* (*n* = 969) versus register-based *University* (*n* = 1636) and *No university* (*n* = 1026)). However, bivariate regression analyses yielded equivalent output: 3.10 higher post-treatment score (95% CI [1.43, 4.76], *p* < .001), for self-reported education as an independent variable, and 3.10 (95% CI [1.44, 4.75], *p* < .001), for the register-based one.

Given that missingness in both sources is the same (*n* = 6), the self-reported measure was used in the full model dictated by ease of data obtainment.

**Marital status**

Clinic-based (self-reported at screening) marital status is a civil status, divided into *Married*, *Single*, *Separated*, and *Widowed*. *Married* includes being officially married as well as de facto married, i.e. in a committed relationship (‘married/living together/living apart together’). For statistical analysis, the variable was dichotomised into *Married* and *Unmarried* based on the discriminatory power.

Register-based marital status, derived from the Longitudinal integrated database for health insurance and labor market studies (LISA), is divided into *Married*, *Not married*, *Divorced*, *Widowed*, *Registered* *partner* (same-sex marriage before 2009), and *Divorced partner* (divorced from same-sex marriage before 2009). The dichotomized variable comprises *Married* (combining *Married* and *Registered* *partner*) and *Unmarried* (combining *Not married*, *Divorced*, *Widowed*, and *Divorced partner*).

The advantage of self-reported marital status is that it captures those who are in a committed relationship but are not officially married, a common constellation in Sweden: the option coded as *Married* is phrased as ʼgift/sambo/särboʼ (‘married/living together/living apart together’) in the screening questionnaire.

Both self-reported and register-based marital status have the same number of missing observations (*n* = 6), albeit in different patients, so missingness cannot be a deciding factor in the source choice. Although the two groups’ composition is very unbalanced (self-reported married 59% versus register-based married 24%), given that the unofficially married patients belong to different groups in the two data sources, the direction and effect size of their association with the outcome are similar (unmarried patients from the self-reported group have on average 3.72 higher post-treatment score (95% CI [2.10, 5.33], *p* < .001), and in the register-based group 4.25 (95% CI [2.41, 6.08], *p* < .001). Hence, it was decided to use the self-reported measure in the full model as it is better equipped to capture meaningful relationships beyond official records.

**Parental status**

Clinic-based (self-reported at screening) parental status was retrieved from the screening question "Do you have children?". Register-based parental status was dichotomized from the number of children recorded in the Total Population Register, based on family ID and civil registration. Notably, 41% of patients self-reported having children, whereas register data shows a higher percentage of 48%. The discrepancy between the two sources stems from the operationalization of parental status in the registers: these data are based on civil registration and thus, tend to include children who reside at the same address but may not be perceived as own children (e.g. partner’s children).

The number of missing values is the same in both sources (*n* = 6). Bivariate analyses showed the same direction (protective) and similar strength of association (weak) but were only statistically significant for the self-reported predictor: having children is associated with 1.66 points lower post-treatment score (95% CI [-3.27, -0.05], *p* = .043), whereas for the register-based predictor, the respective estimates are 1.03 (95% CI [-2.63, -0.57], *p* = .209). These results may reflect the importance of having children as a predictor of treatment outcome only when patients view children as their own. Thus, the self-reported measure was used in the full model.

—

Despite some discrepancies, for all the variables available as both clinic-based and register-based, the association with the outcome of interest was similar suggesting that the available sources can be used interchangeably depending on data availability.

**S2 Table.** Summary of missing data for variables in the analytic sample

| **Variables** | **Missingness** |
| --- | --- |
| **Clinic-based data** |  |
| Post-treatment symptom severity | 524 (19.6%) |
| Pre-treatment symptom severity | 3 (0.1%) |
| Comorbidities | 98 (3.7%) |
| Family history | 101 (3.8%) |
| Education | 6 (0.2%) |
| Marital status | 6 (0.2%) |
| Parental status | 6 (0.2%) |
| Sex | 1 (<0.1%) |
| **Genetic data** |  |
| PRS MDD | 456 (17.1%) |
| PRS ASD | 456 (17.1%) |
| PRS ADHD | 456 (17.1%) |
| PRS Bipolar | 456 (17.1%) |
| PRS Education | 456 (17.1%) |
| PRS IQ | 456 (17.1%) |
| PRS SCZ | 456 (17.1%) |
| **Register-based data** |  |
| Employment | 1 (<0.1%) |
| Income | 6 (0.2%) |
| Any financial benefits | 6 (0.2%) |
| Prior psychiatric diagnosis | 0 (0.0%) |
| Prior medication | 0 (0.0%) |

**S3 Table.** Full list of predictors grouped by data source

1. **Clinic-based predictors**

| **Predictor** | **Variable type** | **Comment** |
| --- | --- | --- |
| Pre-treatment symptom severity | Numeric | Numeric harmonized score combining  all the diagnoses |
| Sex | Binary |  |
| Comorbidities | Binary |  |
| Family history | Binary |  |
| Education | Binary | University/No university |
| Marital status | Binary |  |
| Parental status | Binary | Only used in the full model |

1. **Genetic predictors**

| **Predictor** | **Variable type** | **Comment** |
| --- | --- | --- |
| PRS MDD | Numeric | Standardized |
| PRS ASD | Numeric | Standardized |
| PRS ADHD | Numeric | Standardized |
| PRS Bipolar disorder | Numeric | Standardized |
| PRS Education | Numeric | Standardized |
| PRS IQ | Numeric | Standardized |
| PRS SCZ | Numeric | Standardized |

1. **Register-based predictors**

| **Predictor** | **Variable type** | **Comment** |
| --- | --- | --- |
| Employment | Binary |  |
| Income | Categorical | Individual’s component of the household income adjusted for family composition, in quintiles |
| Any financial benefits | Binary |  |
| Any prior psychiatric diagnosis | Binary | Excluded from multiple regression |
| F1 (Mental and behavioral disorders  due to psychoactive substance use) | Binary | Not the same diagnoses as ICBT treatment, hence no 1-month blanking applied |
| F3 (Mood [affective] disorders) | Binary | - Bipolar disorder is excluded and treated as a separate variable to avoid conflation with MDD  - Same diagnoses as ICBT treatment, hence 1-month blanking applied |
| F4 (Anxiety, dissociative, stress-related, somatoform and other nonpsychotic  mental disorders) | Binary | Same diagnoses as ICBT treatment, hence 1-month blanking applied |
| F5 (Behavioral syndromes associated  with physiological disturbances and  physical factors) | Binary | Not the same diagnoses as ICBT treatment, hence no 1-month blanking applied |
| F6 (Disorders of adult personality  and behavior) | Binary | Not the same diagnoses as ICBT treatment, hence no 1-month blanking applied |
| ASD | Binary | Not limited to pre-treatment diagnosis |
| ADHD | Binary | Not limited to pre-treatment diagnosis |
| Any prior psychotropic medication | Binary | Excluded from multiple regression |
| N06A (Antidepressants) | Binary | Excluded from multiple regression |
| N06AX12 (Bupropion) | Binary | 1-month blanking applied |
| N06AX21 (Duloxetine) | Binary | 1-month blanking applied |
| N06AB03 (Fluoxetine) | Binary | 1-month blanking applied |
| N06AX16 (Venlafaxine) | Binary | 1-month blanking applied |
| N06AX11 (Mirtazapine) | Binary | 1-month blanking applied |
| N06AA09 (Amitriptyline) | Binary | 1-month blanking applied |
| N06AB06 (Sertraline) | Binary | 1-month blanking applied |
| N06AB10 (Escitalopram) | Binary | 1-month blanking applied |
| N06AB04 (Citalopram) | Binary | 1-month blanking applied |
| N06AA04 (Clomipramine) | Binary | 1-month blanking applied |
| N06AB05 (Paroxetine) | Binary | 1-month blanking applied |
| N05B (Anxiolytics) | Binary | Excluded from multiple regression |
| N05BE01 (Buspirone) | Binary | 1-month blanking applied |
| N05BA12 (Alprazolam) | Binary | 1-month blanking applied |
| N05BB01 (Hydroxyzine) | Binary | 1-month blanking applied |
| N05BA01 (Diazepam) | Binary | 1-month blanking applied |
| N05BA04 (Oxazepam) | Binary | 1-month blanking applied |
| N05C (Hypnotics and sedatives) | Binary | Excluded from multiple regression |
| N05CH01 (Melatonin) | Binary | 1-month blanking applied |
| N05CF02 (Zolpidem) | Binary | 1-month blanking applied |
| N05CF01 (Zopiclone) | Binary | 1-month blanking applied |
| N05CM06 (Propiomazine) | Binary | 1-month blanking applied |
| (N05A) Antipsychotics | Binary | 1-month blanking applied |

**Variables with very low variance* (excluded as individual predictors but retained within composite variables):**

1. F0 diagnoses (Mental disorders due to known physiological conditions)
2. F2 diagnoses (Schizophrenia, schizotypal, delusional, and other non-mood psychotic disorders)
3. F7 diagnoses (Intellectual Disabilities)
4. F8 diagnoses (Pervasive and specific developmental disorders), excluding ASD
5. F9 diagnoses (Behavioral and emotional disorders with onset usually occurring in childhood and adolescence), excluding ADHD
6. F99 diagnoses (Unspecified mental disorder)
7. Diagnosis of bipolar affective disorder
8. Number of sick leave days
9. Receipt of rehabilitation compensation (rehabiliteringsersättning)
10. Duration of receipt of rehabilitation compensation
11. Duration of receipt of unemployment benefit (arbetslöshetsersättning)
12. Receipt of compensation for participation in active labor market policies (ALMPs)
13. Duration of receipt of ALMPs

Outtake of antidepressants (N06A):

1. N06AA10
2. N06AA21
3. N06AB08
4. N06AF03
5. N06AF04
6. N06AG02
7. N06AX03
8. N06AX05
9. N06AX18
10. N06AX22
11. N06AX26

Outtake of anxiolytics (N05B):

1. N05BA06

Outtake of hypnotics and sedatives (N05C):

1. N05CD02
2. N05CD03
3. N05CD05
4. N05CD08
5. N05CF03
6. N05CM02
7. N05CM09

Outtake of antipsychotics (N05A):

1. N05AA02
2. N05AB01
3. N05AB03
4. N05AB04
5. N05AD01
6. N05AD03
7. N05AE04
8. N05AF01
9. N05AF03
10. N05AF05
11. N05AH03
12. N05AN01
13. N05AX08
14. N05AX12

*<1% frequency of the second value and percentage of unique values of <10% of the total number of data points

**Sensitivity analysis of outcome missingness**

Missingness of outcome values was partly associated with observed values in some other variables, namely Education, Sex, Family history of psychopathology, Parental status, Age, and Prior psychiatric diagnoses. Patients with missing outcome were less likely to have a university degree (58% vs 65% among non-missing, χ2 (1, 2668) = 8.717, *p* < .01), more likely to be male (44% vs 37%, χ2 (1, 2668) = 9.4844, *p* < .01), more likely to have a family history of psychopathology (75% vs 70%, χ2 (1, 2668) = 9.4844, *p* = .032), less likely to have children (35% vs 43%, χ2 (1, 2668) = 11.344, *p* < .001), more likely to be younger (mean age 33.3 vs 36.2, t(843.62) = 5.36, *p* < .001), and more likely to have a prior psychiatric diagnosis (21% vs 13%, χ2 (1, 2668) = 2.311, *p* < .001).

**S4 Table.** Detailed descriptive statistics for all disorders

| **Variable** | **Total**  (*n* = 2668) | **MDD**  (*n* = 1300) | **PD**  (*n* = 727) | **SAD**  (*n* = 641) |
| --- | --- | --- | --- | --- |
| Pre-treatment symptom severity, mean (SD) | 47.6 (17.8) | 51.6 (15.7) | 40.2 (18.8) | 47.7 (17.9) |
| Post-treatment symptom severity, mean (SD) | 28.2 (18.8) | 30.3 (18.7) | 17.8 (16.1) | 35.3 (17.2) |
| Relative symptom change, % (SD) | -41.3 (37.9) | -41.7 (34.3) | -53.4 (47.0) | -28.1 (26.5) |
| Age, mean (SD) | 35.6 (11.4) | 37.6 (11.9) | 34.6 (10.8) | 32.7 (10.3) |
| Male sex, *n* (%) | 1014 (38.0%) | 443 (34.1%) | 292 (40.2%) | 279 (43.6%) |
| Psychiatric comorbidities, *n* (%) | 862 (33.5%) | 394 (31.4%) | 265 (37.9%) | 203 (32.9%) |
| Family history of psychopathology, *n* (%) | 1812 (70.6%) | 888 (71%) | 488 (69.7%) | 436 (70.7%) |
| Self-reported married, *n* (%) | 1566 (58.8%) | 727 (56.1%) | 477 (65.7%) | 362 (56.7%) |
| Self-reported having children, *n* (%) | 1102 (41.4%) | 603 (46.4%) | 302 (41.5%) | 199 (31.0%) |
| Self-reported university education, *n* (%) | 1693 (63.6%) | 890 (68.8%) | 406 (55.9%) | 397 (62.1%) |
| Register-based employed, *n* (%) | 2448 (91.8%) | 1203 (92.9%) | 674 (92.8%) | 571 (89.1%) |
| Annual income (SEK), mean (SD) | 241,713 (201,190) | 256,187 (202,155) | 244,258 (232,968) | 209,591 (150,742) |
| Financial benefits in the past year, *n* (%) | 556 (20.9%) | 302 (23.3%) | 148 (20.4%) | 106 (16.5%) |
| Prior psychiatric diagnosis, *n* (%) | 1793 (67.2%) | 895 (68.8%) | 518 (71.3%) | 380 (59.3%) |
| Prior F1 | 188 (7.0%) | 92 (7.1%) | 48 (6.6%) | 48 (7.5%) |
| Prior F3 (excl. BPAD) | 978 (36.7%) | 669 (51.5%) | 144 (19.8%) | 165 (25.7%) |
| Prior F4 | 1350 (50.6%) | 557 (42.8%) | 477 (65.5%) | 316 (49.3%) |
| Prior F5 | 251 (9.4%) | 144 (11.1%) | 48 (6.6%) | 59 (9.2%) |
| Prior F6 | 47 (1.8%) | 20 (1.5%) | 13 (1.8%) | 14 (2.2%) |
| ASD | 41 (1.5%) | 22 (1.7%) | 4 (0.6%) | 15 (2.3%) |
| ADHD | 133 (5.0%) | 69 (5.3%) | 26 (3.6%) | 38 (5.9%) |
| Prior psychotropic medication, *n* (%) | 1665 (62.4%) | 863 (66.4%) | 472 (64.9%) | 330 (51.5%) |
| Antidepressants | 1349 (50.6%) | 749 (57.6%) | 329 (45.3%) | 271 (42.3%) |
| Bupropion | 95 (3.6%) | 61 (4.7%) | 11 (1.5%) | 23 (3.6%) |
| Duloxetine | 88 (3.3%) | 55 (4.2%) | 15 (2.1%) | 18 (2.8%) |
| Fluoxetine | 149 (5.6%) | 87 (6.7%) | 33 (4.5%) | 29 (4.5%) |
| Venlafaxine | 166 (6.2%) | 106 (8.2%) | 32 (4.4%) | 28 (4.4%) |
| Mirtazapine | 201 (7.5%) | 119 (9.2%) | 40 (5.5%) | 42 (6.6%) |
| Amitriptyline | 65 (2.4%) | 36 (2.8%) | 16 (2.2%) | 13 (2.0%) |
| Sertraline | 582 (21.8%) | 310 (23.8%) | 139 (19.1%) | 133 (20.7%) |
| Escitalopram | 275 (10.3%) | 161 (12.4%) | 54 (7.4%) | 60 (9.4%) |
| Citalopram | 481 (18.0%) | 280 (21.5%) | 122 (16.8%) | 79 (12.3%) |
| Clomipramine | 30 (1.1%) | 9 (0.7%) | 16 (2.2%) | 5 (0.8%) |
| Paroxetine | 91 (3.4%) | 38 (2.9%) | 36 (5.0%) | 17 (2.7%) |
| Anxiolytics | 1025 (38.4%) | 461 (35.5%) | 360 (49.5%) | 204 (31.8%) |
| Buspirone | 32 (1.2%) | 19 (1.5%) | 3 (0.4%) | 10 (1.6%) |
| Alprazolam | 63 (2.4%) | 18 (1.4%) | 27 (3.7%) | 18 (2.8%) |
| Hydroxyzine | 758 (28.4%) | 344 (26.5%) | 259 (35.6%) | 155 (24.2%) |
| Diazepam | 111 (4.2%) | 59 (4.5%) | 38 (5.2%) | 14 (2.2%) |
| Oxazepam | 407 (15.3%) | 174 (13.4%) | 161 (22.1%) | 72 (11.2%) |
| Hypnotics and sedatives | 703 (26.3%) | 409 (31.5%) | 166 (22.8%) | 128 (20.0%) |
| Melatonin | 74 (2.8%) | 36 (2.8%) | 11 (1.5%) | 27 (4.2%) |
| Zolpidem | 236 (8.8%) | 148 (11.4%) | 46 (6.3%) | 42 (6.6%) |
| Zopiclone | 376 (14.1%) | 236 (18.2%) | 78 (10.7%) | 62 (9.7%) |
| Propiomazine | 334 (12.5%) | 195 (15.0%) | 80 (11.0%) | 59 (9.2%) |
| Antipsychotics | 92 (0.3%) | 45 (3.5%) | 25 (3.4%) | 22 (3.4%) |

**S5 Table.** Descriptive statistics for the observed, imputed, and complete data*

| **Variable** | **Observed** | **Imputed** | **Complete** |
| --- | --- | --- | --- |
| Pre-treatment symptom severity, mean (SD) | 47.6 (17.8) | 54.6 (13.2) | 47.6 (17.8) |
| Post-treatment symptom severity, mean (SD) | 28.2 (18.8) | 34.8 (22.1) | 29.5 (19.7) |
| Psychiatric comorbidities, *n* (%) | 862 (33.5%) | 32 (32.5%) | 894 (33.5%) |
| Family history of psychopathology, *n* (%) | 1812 (70.6%) | 71 (72.3%) | 1885 (70.7%) |
| Self-reported married, *n* (%) | 1566 (58.8%) | 3 (50.0%) | 1569 (58.8%) |
| Self-reported having children, *n* (%) | 1102 (41.4%) | 1 (20.0%) | 1103 (41.3%) |
| Self-reported university education, *n* (%) | 1693 (63.6%) | 3 (50.0%) | 1696 (63.6%) |
| Register-based employed, *n* (%) | 2448 (92.0%) | 5 (80.0%) | 2453 (91.9%) |
| Financial benefits in the past year, *n* (%) | 556 (20.9%) | 2 (40.0%) | 558 (20.9%) |

*Age, Sex, Annual income, Prior psychiatric diagnoses, and Prior psychotropic medication were not imputed due to no missingness

*Observed, non-missing values*

*Imputed, missing values that were derived in the imputation process by pooling estimates from the 20 imputations*

*Complete, summary statistics for the full dataset combining observed and imputed values used in all analyses*

**S6 Table.** Results of multiple regression on a complete case dataset with post-treatment symptom severity as the outcome

| **Predictor** | **Estimate (95% CI)** | ***p*-value** |
| --- | --- | --- |
| Pre-treatment score | 0.52 (0.47, 0.56) | <.001*** |
| Comorbidities | -0.15 (-1.78, 1.47) | .854 |
| Family history | 0.21 (-1.45, 1.87) | .808 |
| Sex (ref: male) | -2.05 (-3.64, -0.47) | .011* |
| Unmarried | 1.44 (-0.16, 3.04) | .077 |
| No children | 0.24 (-1.49, 1.97) | .785 |
| No university | 1.37 (-0.22, 2.97) | .091 |
| Unemployed | 2.77 (-0.21, 5.74) | .069 |
| Income (quintiles) | -0.84 (-1.46, -0.22) | .008** |
| Any financial benefits | 1.93 (-0.04, 3.90) | .054 |
| PRS MDD | 0.44 (-0.36, 1.25) | .277 |
| PRS ASD | 0.51 (-0.28, 1.29) | .207 |
| PRS ADHD | 0.02 (-0.80, 0.84) | .963 |
| PRS BPAD | -0.54 (-1.37, 0.29) | .203 |
| PRS Education | 0.32 (-0.55, 1.20) | .469 |
| PRS IQ | 0.27 (-0.60, 1.13) | .544 |
| PRS SCZ | 0.44 (-0.39, 1.27) | .302 |
| Prior psychiatric diagnosis |  |  |
| Prior F1 | -2.78 (-6.00, 0.44) | .091 |
| Prior F3 (excl. BPAD) | 2.47 (0.61, 4.34) | .009** |
| Prior F4 | 0.22 (-1.44, 1.88) | .794 |
| Prior F5 | 3.04 (0.11, 5.97) | .042* |
| Prior F6 | 6.48 (0.67, 12.29) | .029* |
| ASD | 8.56 (2.32, 14.81) | .007** |
| ADHD | 6.64 (2.75, 10.53) | <.001*** |
| Prior medication |  |  |
| Antidepressants |  |  |
| Bupropion | 6.91 (2.49, 11.33) | .002** |
| Duloxetine | 0.30 (-4.18, 4.79) | .894 |
| Fluoxetine | 3.79 (0.24, 7.34) | .037* |
| Venlafaxine | 2.96 (-0.47, 6.39) | .090 |
| Mirtazapine | -1.26 (-4.46, 1.95) | .441 |
| Amitriptyline | 2.63 (-2.13, 7.38) | .278 |
| Sertraline | 1.80 (-0.20, 3.79) | .078 |
| Escitalopram | 1.44 (-1.17, 4.06) | .279 |
| Citalopram | 2.18 (0.07, 4.28) | .043* |
| Clomipramine | -0.69 (-7.90, 6.51) | .850 |
| Paroxetine | -0.17 (-4.26, 3.93) | .936 |
| Anxiolytics |  |  |
| Buspirone | 2.54 (-4.96, 10.04) | .506 |
| Alprazolam | 2.92 (-2.39, 8.22) | .282 |
| Hydroxyzine | -1.60 (-3.47, 0.27) | .094 |
| Diazepam | -0.83 (-4.63, 2.98) | .670 |
| Oxazepam | -0.99 (-3.32, 1.35) | .408 |
| Hypnotics and sedatives |  |  |
| Melatonin | 2.12 (-2.97, 7.20) | .414 |
| Zolpidem | 1.37 (-1.49, 4.22) | .348 |
| Zopiclone | 0.20 (-2.27, 2.66) | .876 |
| Propiomazine | -1.50 (-4.09, 1.10) | .258 |
| Antipsychotics | -2.66 (-7.06, 1.75) | .237 |

*PRS, polygenic risk score; MDD, Major depressive disorder; ASD, Autism spectrum disorder; ADHD, Attention deficit hyperactivity disorder; BPAD, Bipolar affective disorder; IQ, intelligence quotient; SCZ, Schizophrenia; F1, Mental and behavioural disorders due to psychoactive substance use; F3, Mood [affective] disorders; F4, Anxiety, dissociative, stress-related, somatoform and other nonpsychotic mental disorders; F5, Behavioural syndromes associated with physiological disturbances and physical factors; F6, Disorders of adult personality and behaviour; CI, confidence interval*

*Significance levels: *p < .05, **p < .01, ***p < .001*

**S7 Table.** Results of multiple regression with symptom change as the outcome

| **Predictor** | **Estimate (95% CI)** | ***p*-value** |
| --- | --- | --- |
| Pre-treatment score | -0.05 (-0.15, 0.05) | .344 |
| Comorbidities | 0.24 (-3.83, 4.31) | .908 |
| Family history | -1.63 (-5.42, 2.16) | .398 |
| Sex (ref: male) | -3.09 (-6.93, 0.75) | .114 |
| Unmarried | 4.67 (0.91, 8.43) | .015* |
| No children | 2.35 (-1.57, 6.27) | .239 |
| No university | 3.20 (-0.64, 7.04) | .102 |
| Unemployed | 6.22 (-0.56, 13.00) | .072 |
| Income (quintiles) | -1.04 (-2.38, 0.31) | .130 |
| Any financial benefits | 5.47 (1.26, 9.69) | .011* |
| PRS MDD | 1.40 (-0.80, 3.60) | .208 |
| PRS ASD | 1.06 (-0.97, 3.08) | .304 |
| PRS ADHD | -0.68 (-3.06, 1.70) | .570 |
| PRS BPAD | -1.17 (-3.32, 0.97) | .281 |
| PRS Education | 0.61 (-1.69, 2.91) | .598 |
| PRS IQ | 0.87 (-1.75, 3.49) | .508 |
| PRS SCZ | 1.18 (-1.12, 3.49) | .310 |
| Prior psychiatric diagnosis |  |  |
| Prior F1 | -0.84 (-9.49, 7.80) | .847 |
| Prior F3 (excl. BPAD) | 2.36 (-1.95, 6.68) | .282 |
| Prior F4 | 2.56 (-1.24, 6.35) | .185 |
| Prior F5 | 6.09 (-0.74, 12.92) | .080 |
| Prior F6 | 9.31 (-7.00, 25.62) | .259 |
| ASD | 18.00 (0.32, 35.68) | .046* |
| ADHD | 14.04 (3.41, 24.67) | .010** |
| Prior medication |  |  |
| Antidepressants |  |  |
| Bupropion | 13.61 (2.04, 25.18) | .022* |
| Duloxetine | 0.25 (-11.68, 12.19) | .966 |
| Fluoxetine | 7.64 (-0.67, 15.95) | .071 |
| Venlafaxine | 7.68 (-0.58, 15.93) | .068 |
| Mirtazapine | -2.60 (-10.29, 5.09) | .505 |
| Amitriptyline | 5.07 (-6.50, 16.64) | .389 |
| Sertraline | 4.56 (-0.12, 9.23) | .056 |
| Escitalopram | 3.58 (-2.12, 9.29) | .217 |
| Citalopram | 7.86 (2.80, 12.92) | .003** |
| Clomipramine | 10.03 (-6.25, 26.32) | .226 |
| Paroxetine | 3.35 (-6.06, 12.77) | .484 |
| Anxiolytics |  |  |
| Buspirone | 12.61 (-7.03, 32.24) | .205 |
| Alprazolam | 4.03 (-8.98, 17.04) | .541 |
| Hydroxyzine | -4.50 (-8.56, -0.44) | .030* |
| Diazepam | -1.21 (-9.91, 7.48) | .784 |
| Oxazepam | -1.27 (-6.26, 3.72) | .617 |
| Hypnotics and sedatives |  |  |
| Melatonin | 3.48 (-8.23, 15.18) | .559 |
| Zolpidem | 2.78 (-4.11, 9.68) | .427 |
| Zopiclone | 1.20 (-4.37, 6.77) | .671 |
| Propiomazine | -4.40 (-10.60, 1.80) | .163 |
| Antipsychotics | -6.08 (-16.83, 4.66) | .265 |

*PRS, polygenic risk score; MDD, Major depressive disorder; ASD, Autism spectrum disorder; ADHD, Attention deficit hyperactivity disorder; BPAD, Bipolar affective disorder; IQ, intelligence quotient; SCZ, Schizophrenia; F1, Mental and behavioural disorders due to psychoactive substance use; F3, Mood [affective] disorders; F4, Anxiety, dissociative, stress-related, somatoform and other nonpsychotic mental disorders; F5, Behavioural syndromes associated with physiological disturbances and physical factors; F6, Disorders of adult personality and behaviour; CI, confidence interval*

*Significance levels: *p < .05, **p < .01, ***p < .001*

**Figure S1.** Density plot of observed and imputed outcome data


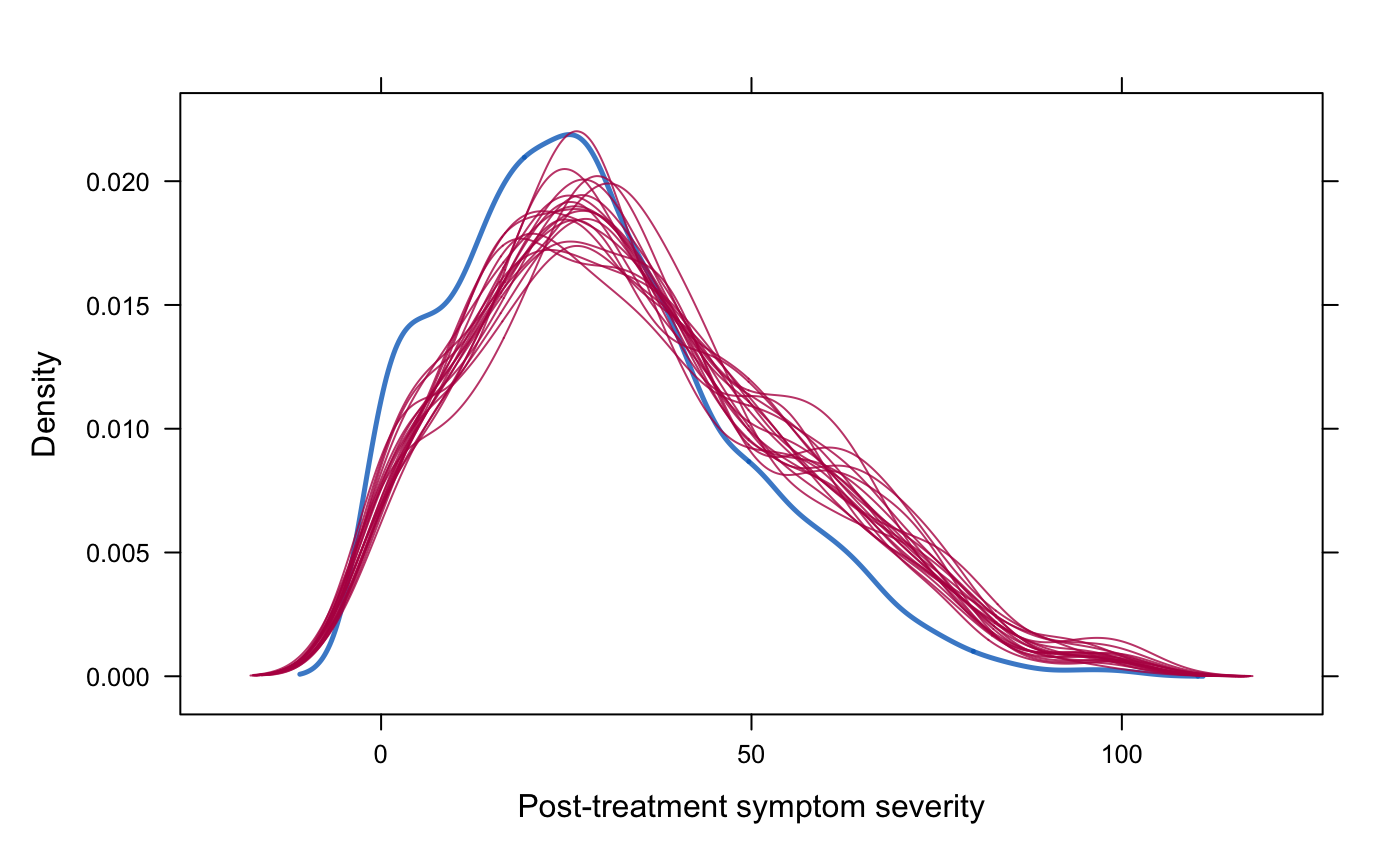


*Blue - observed values; Red - imputed values from m = 20 imputed datasets*

Aligned with the observed pattern of missingness, post-imputation diagnostics revealed that the multiply imputed outcome values (*red*) were significantly higher than the observed ones (*blue*), which does not constitute a problem but rather highlights the issue that the imputation is called to solve^^[[1]](#footnote-1)^^. Moreover, this discrepancy was reduced in the complete dataset comprising both observed and imputed data, with the Kolmogorov-Smirnov test yielding *p* = .126, which indicates that the two samples were taken from the same distribution (a p-value of ≤ .05 would suggest a significant difference between the distribution of the observed and imputed data, indicating potential issues with the imputation process). The mean (SD) of the outcome variable in the complete analytic sample is 29.5 (19.7), slightly higher than the observed mean (SD) of 28.2 (18.8), thus correcting for the bias toward the null.

1. Stuart EA, Azur M, Frangakis C, Leaf P. Multiple imputation with large data sets: a case study of the Children's Mental Health Initiative. *Am J Epidemiol* 2009; 169(9): 1133-1139 <https://doi.org/10.1093/aje/kwp026> [↑](#footnote-ref-1)
